# Connecting the Dots: FGF21 as a Potential Link between Obesity and Cardiovascular Health in Acute Coronary Syndrome Patients

**DOI:** 10.3390/cimb46080501

**Published:** 2024-08-03

**Authors:** Cristina Elena Negroiu, Anca-Lelia Riza, Ioana Streață, Iulia Tudorașcu, Cristina Maria Beznă, Adrian Ionuț Ungureanu, Suzana Dănoiu

**Affiliations:** 1 Department of Pathophysiology, University of Medicine and Pharmacy of Craiova, 200642 Craiova, Romania; irtudorascu@gmail.com (I.T.); bezna.mariacristina@gmail.com (C.M.B.); suzanadanoiu@yahoo.com (S.D.); 2 Doctoral School, University of Medicine and Pharmacy of Craiova, 200349 Craiova, Romania; adrianungureanu90@yahoo.com; 3 Laboratory of Human Genomics, University of Medicine and Pharmacy of Craiova, 200638 Craiova, Romania; ioana.streata@umfcv.ro; 4 Regional Centre of Medical Genetics Dolj, Emergengy County Hospital Craiova, 200642 Craiova, Romania; 5 Department of Cardiology, County Clinical Emergency Hospital of Craiova, 200642 Craiova, Romania

**Keywords:** obesity, FGF21, myocardial infarction

## Abstract

Fibroblast growth factor 21 (FGF21) is a hormone involved in regulating the metabolism, energy balance, and glucose homeostasis, with new studies demonstrating its beneficial effects on the heart. This study investigated the relationship between FGF21 levels and clinical, biochemical, and echocardiographic parameters in patients with acute coronary syndromes (ACSs). This study included 80 patients diagnosed with ACS between May and July 2023, categorized into four groups based on body mass index (BMI): Group 1 (BMI 18.5–24.9 kg/m^2^), Group 2 (BMI 25–29.9 kg/m^2^), Group 3 (BMI 30–34.9 kg/m^2^), and Group 4 (BMI ≥ 35 kg/m^2^). Serum FGF21 levels were measured by ELISA (Abclonal Catalog NO.: RK00084). Serum FGF21 levels were quantifiable in 55 samples (mean ± SD: 342.42 ± 430.17 pg/mL). Group-specific mean FGF21 levels were 238.98 pg/mL ± SD in Group 1 (n = 14), 296.78 pg/mL ± SD in Group 2 (n = 13), 373.77 pg/mL ± SD in Group 3 (n = 12), and 449.94 pg/mL ± SD in Group 4 (n = 16), with no statistically significant differences between groups (*p* = 0.47). Based on ACS diagnoses, mean FGF21 levels were 245.72 pg/mL for STEMI (n = 21), 257.89 pg/mL for NSTEMI (n = 9), and 456.28 pg/mL for unstable angina (n = 25), with no significant differences observed between these diagnostic categories. Significant correlations were identified between FGF21 levels and BMI, diastolic blood pressure, and serum chloride. Regression analyses revealed correlations with uric acid, chloride, and creatinine kinase MB. This study highlights the complex interplay between FGF21, BMI, and acute coronary syndromes. While no significant differences were found in FGF21 levels between the different BMI and ACS diagnostic groups, correlations with clinical and biochemical parameters suggest a multifaceted role of FGF21 in cardiovascular health. Further research with a larger sample size is warranted to elucidate these relationships.

## 1. Introduction

Obesity has emerged as a significant global health challenge in recent decades, particularly in high-income countries where its prevalence has risen dramatically. Obesity, smoking, high blood pressure, and high cholesterol pose high risks for cardiovascular diseases (CVDs) such as acute coronary syndromes [1].

Acute coronary syndromes (ACSs) represent a critical spectrum of cardiovascular diseases that include unstable angina (UA), non-ST-elevation myocardial infarction (NSTEMI), and ST-elevation myocardial infarction (STEMI) [2]. These conditions are typically precipitated by the rupture or erosion of atherosclerotic plaques in coronary arteries, leading to partial or complete obstruction of blood flow to the heart muscle. ACS is often the initial manifestation of coronary artery disease (CAD) and is a major contributor to morbidity and mortality worldwide [3].

FGF21 is a metabolic hormone that regulates energy homeostasis and metabolism, first identified in 2000 by Nishimura and collaborators [4]. It plays a crucial role in glucose and lipid metabolism and adapting to metabolic stressors such as fasting and exercise [5]. FGF21 has several beneficial effects, including enhancing glucose uptake into adipose tissues, reducing serum hyperglycemia, stimulating free fatty acid oxidation, lowering LDL-cholesterol levels, inhibiting liver gluconeogenesis and lipogenesis, improving insulin sensitivity, and regulating the secretion of other hormones, e.g., adiponectin, to control energy metabolism [5].

FGF21 is secreted primarily by the liver, but many other tissues, such as white adipose tissue, brown adipose tissue, skeletal muscles, the pancreas, and others, are also involved in its secretion [6,7,8]. Unlike other members of the fibroblast growth factor family, FGF 21 requires the β-Klotho (KLB) coreceptor to exert its effects. Initial studies showed that the β-Klotho (KLB) coreceptor is modestly expressed in the heart, leading to the belief that the effects of FGF21 on the heart were minimal [9]. However, recent research has demonstrated that FGF21 plays a key role in cardiac remodeling [10].

Preclinical studies have highlighted the beneficial effects of FGF21 in various cardiac models, demonstrating its protective role against conditions such as myocardial hypertrophy, ischemia, dilated cardiomyopathy, and ischemia/reperfusion injury [11,12,13,14]. These studies show that FGF21 provides therapeutic benefits by reducing apoptosis, oxidative stress, and inflammation while improving energy supply [11].

In the clinical setting, FGF21 levels are elevated in a range of human diseases, including obesity [15,16,17,18], non-alcoholic fatty liver disease [18,19,20,21], diabetes mellitus [22], chronic kidney disease [23,24], atherosclerosis [25,26], and coronary heart disease [27,28]. Increased FGF21 levels may indicate the body’s response to metabolic stress, which could be a target for treating metabolic disorders and cardiovascular diseases.

This study focused on measuring FGF21 levels in patients diagnosed with ACS and analyzing these levels concerning body mass index (BMI) categories. Specifically, we aimed to determine if FGF21 levels correlate with BMI and to explore how FGF21 values vary across different types of ACS. Additionally, the study sought to examine the relationship between FGF21 levels and various anthropometric, clinical, biochemical, and echocardiographic parameters in these patients. Our primary hypothesis is that serum FGF21 levels are correlated with BMI.

## 2. Materials and Methods

### 2.1. Study Participants

**Figure 1:**
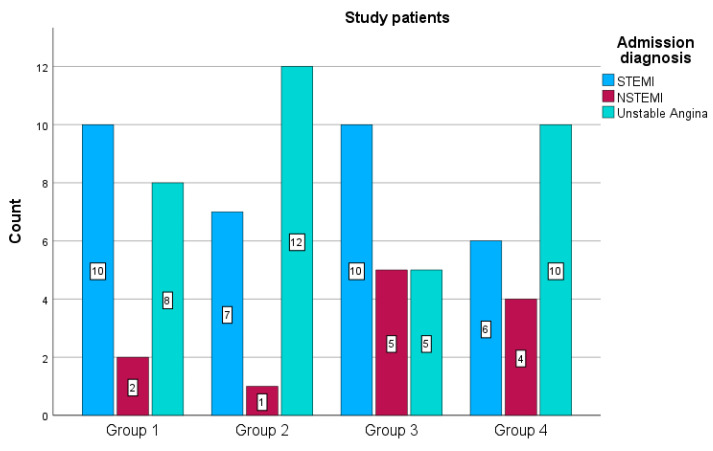
Distribution of Admission Diagnoses across BMI Groups in Study Patients.

The study enrolled 80 patients (32 females, 48 males). Participants were recruited between May and July 2023 from the Cardiology Clinic of the Emergency County Hospital Craiova, Romania. Of the 80 patients with ACS, 33 had STEMI, 12 had NSTEMI, and 35 had unstable angina. The study groups were as follows: (1) Group 1 includes 20 patients with BMI between 18.5 and 24.9 kg/m^2^, (2) Group 2 includes 20 patients with BMI between 25 and 29.9 kg/m^2^, (3) Group 3 includes 20 patients with BMI between 30 and 34.9 kg/m^2^, and (4) Group 4 includes 20 patients with BMI more than 35 kg/m^2^. Group 1 had 10 STEMI, 2 NSTEMI, and 8 unstable angina cases. Group 2 had 7 STEMI, 1 NSTEMI, and 12 unstable angina cases. Group 3 had 10 STEMI, 5 NSTEMI, and 5 unstable angina cases. Group 4 had 6 STEMI, 4 NSTEMI, and 10 unstable angina cases. For simplicity, this distribution can also be depicted in Figure 1.

### 2.2. Inclusion Criteria

An age between 40 and 75, admission diagnosis: acute coronary syndromes, and patients who provided informed consent. ***Exclusion Criteria:*** active pregnancy, ongoing cancer, immunological disorders, myopathies or systemic diseases affecting muscle tissue, and surgical interventions conducted within the last six months.

### 2.3. Ethical Considerations

The study was conducted according to the Declaration of Helsinki and approved by the Ethics and Scientific University Deontology Committee of the University of Medicine and Pharmacy Craiova, no. 91/27.04.2023.

### 2.4. Anthropometric Measurements and Biological Constants

Anthropometric measurements included body weight, height, and abdominal circumference. Body mass index was calculated as weight divided by height squared. Blood samples were collected from the participant between 8:00 am and 10:00 am after an overnight fast process and stored for subsequent analysis. FGF21 was determined by enzyme-linked immunosorbent assay (ELISA) according to the manufacturer’s protocol (Abclonal Catalog NO.: RK00084). The kit has an intra-assay coefficient of variation (CV) < 10% and an inter-assay CV < 15%. Standard clinical laboratory techniques were employed for routine biochemical investigations such as complete blood count, erythrocyte sedimentation rate, urea, creatinine, glucose, and uric acid. The lipid profile was determined using standardized enzymatic colorimetric tests. All these measurements were conducted by the hospital laboratory, ensuring adherence to standardized protocols and quality control.

### 2.5. Additional Information

Transthoracic echocardiography was performed on each patient, extracting data on the cardiac chamber wall size, cavity dimensions, systolic function (ejection fraction), and diastolic function. A coronary angiography was conducted on 75 of the 80 patients, and results were extracted for study completion.

### 2.6. Statistical Analysis

All statistical analyses were conducted using IBM SPSS Statistics 29.0.0.0 (IBM Corporation, Armonk, NY, USA). Results are presented as mean ± standard deviation. Continuous variables were tested for normality using the Shapiro–Wilk test to determine if the data followed a normal distribution. Group comparisons were initially conducted using one-way ANOVA to detect significant differences among the four groups categorized by BMI. This was followed by post hoc Tukey HSD tests. Correlations between variables were analyzed using Kendall’s tau correlation coefficient. Simple regression analysis was used to model the relationship between independent and dependent variables. Statistical significance was set at a *p*-value of <0.05 for all tests conducted.

A power analysis was conducted using G*Power 3.1.9.4 software to determine the appropriate sample size. The settings for an omnibus ANOVA test assumed a large effect size (f = 0.70), alpha error probability of 0.10, and power of 0.70. This indicated that 80 participants (20 per group) were required to detect significant differences.

## 3. Results

Our study included 80 patients, divided into four groups based on BMI, with 20 participants in each group. Table 1 summarizes the details of the study participants grouped by BMI. Our analysis of FGF21 levels across different BMI categories revealed that for Group 1, the mean FGF21 level was 238.98 pg/mL ± SD, based on 14 samples. In Group 2, the mean FGF21 level was 296.78 pg/mL ± SD, derived from 13 samples. The mean FGF21 level in Group 3 was 373.77 pg/mL ± SD, with data from 12 samples. Finally, in Group 4, the highest mean FGF21 level was observed at 449.94 pg/mL ± SD from 16 samples. Despite the observed increase in FGF21 levels with higher BMI categories, statistical analysis indicated no significant differences between the groups, with a *p*-value of 0.47.

**Figure 2:**
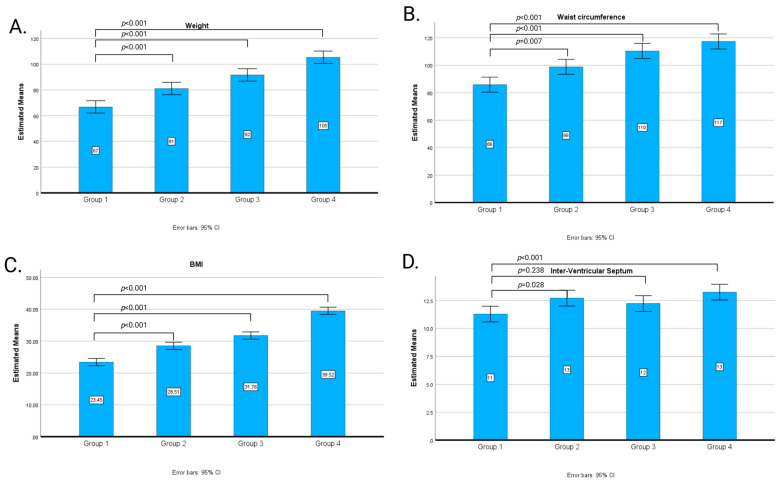

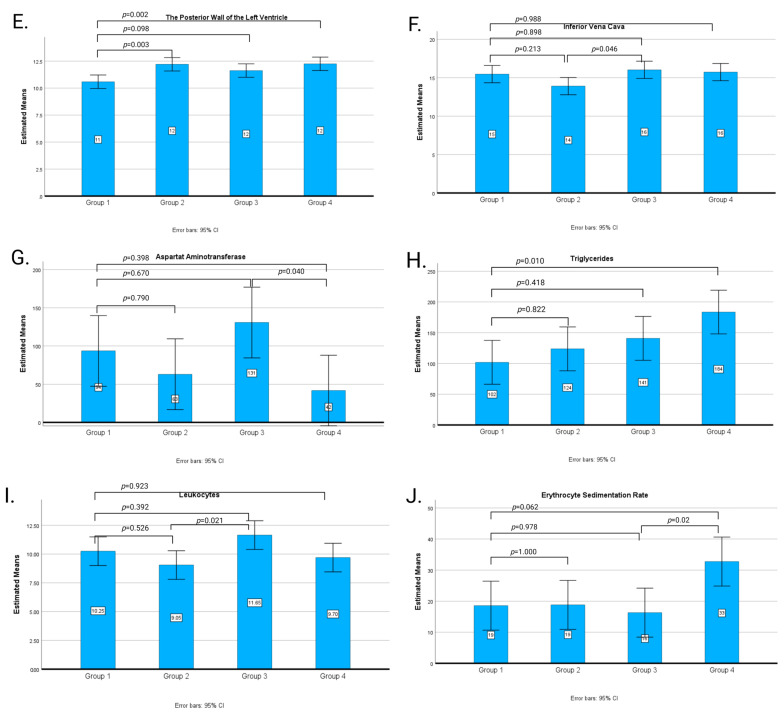
Parameters with significant differences among the study groups: Group 1 includes 20 patients with a BMI between 18.5 and 24.9 kg/m^2^, (2) Group 2 includes 20 patients with a BMI between 25 and 29.9 kg/m^2^, (3) Group 3 includes 20 patients with a BMI between 30 and 34.9 kg/m^2^, and (4) Group 4 includes 20 patients with a BMI greater than 35 kg/m^2^. The panels show the comparison across groups for (**A**) weight, (**B**) waist circumference, (**C**) BMI, (**D**) interventricular septum thickness, (**E**) posterior wall thickness of the left ventricle, (**F**) inferior vena cava diameter, (**G**) aspartate aminotransferase levels, (**H**) triglycerides levels, (**I**) leukocytes and (**J**) erythrocyte sedimentation rate. Post hoc analysis was conducted to identify significant differences between the groups. Statistically significant differences (*p* < 0.05) are indicated. Error bars represent the standard deviation (SD).

The application of one-way ANOVA to compare the four groups revealed a statistically significant difference in weight, waist circumference, BMI, interventricular septum, the posterior wall of the left ventricle, inferior vena cava, aspartate aminotransferase, triglycerides, leukocytes, and erythrocyte sedimentation rate. Post hoc analysis was conducted to determine the significant differences between groups. These results can be easily observed in Figure 2.

**Figure 3:**
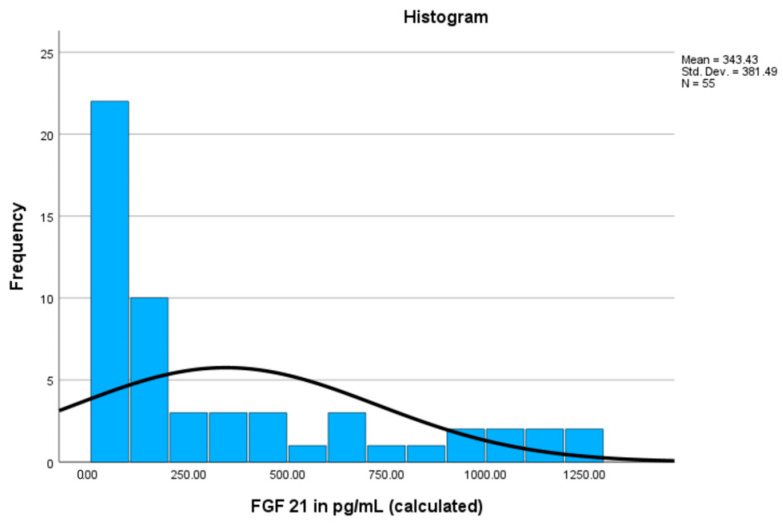
The FGF21 values in pg/mL in the conducted study.

Serum FGF21 levels were measurable in 55 samples using our method (min = 33.7 pg/mL, max = 1278 pg/mL, mean = 342.42), while the remaining samples were either above measuring thresholds (10%, 8 cases) or below (21.25%, 17 cases). These results are highlighted in Figure 3. Among these samples, 21 patients (38.18%) were diagnosed with STEMI, exhibiting a mean FGF21 level of 245.72 pg/mL. In the group of patients diagnosed with NSTEMI, which included nine individuals (16.36%), the mean FGF21 level was 257.89 pg/mL. The largest subgroup consisted of 25 patients (45.45%) with unstable angina, where the mean FGF21 level was notably higher at 456.28 pg/mL. Despite the higher mean levels observed in this group, the overall statistical analysis revealed no significant differences in FGF21 levels between the diagnostic groups. These results are shown in Table 2.

The correlations between FGF21 levels and parameters were analyzed using Kendall’s tau-b coefficient. The results showed weak but statistically significant correlations for BMI (τ = 0.193, *p* = 0.038), diastolic blood pressure (τ = 0.214, *p* = 0.034), and chloride levels (τ = 0.269, *p* = 0.025). In Table 3, we can identify only the parameters that correlate with FGF 21. These findings suggest that higher FGF21 levels are modestly associated with higher BMI, diastolic blood pressure, and chloride levels.

Linear regression analyses were conducted to predict FGF21 based on various variables. Among these analyses, significant regression equations were identified for uric acid (F(1, 53) = 5.208, *p* = 0.027, explaining 8% of the variance, R^2^ = 0.089), chloride (F(1, 34) = 5.048, *p* = 0.031, explaining 12% of the variance, R^2^ = 0.129), and creatine kinase (F(1, 10) = 7.857, *p* = 0.019, explaining 44% of the variance, R^2^ = 0.44), indicating robust associations only in these three instances. The results obtained through linear regression analyses are schematically presented in Table 4.

## 4. Discussion

Regarding FGF21 levels, median values for healthy individuals typically range between 100 and 200 pg/mL, although variations can occur [27]. The study of Michaela Keuper and colleagues discovered that the distribution of FGF21 serum concentrations can be positively skewed, indicating a small subset of individuals with exceptionally high FGF21 levels, causing the distribution to shift to the right. Additionally, they noted instances where FGF21 levels fell below the detection limit of conventional ELISA tests [27]. Our study revealed considerable variability in serum FGF21 levels, with a subset of participants exhibiting exceptionally high concentrations, contributing to the observed positive skewness. Additionally, confirming challenges with ELISA detection limits, as indicated in the literature, our study encountered instances where FGF21 levels fell below the detection limit, emphasizing the complexities in accurately assessing lower and higher concentrations in this cohort.

Although our study did not find a significant difference in FGF21 levels across the four BMI-defined groups, a distinct trend emerged showing increased FGF21 levels with increasing BMI. Previous research has established that FGF21 is a significant marker for obesity, as it often correlates with the degree of adiposity and the success of weight loss efforts [28,29]. FGF21’s involvement in metabolic processes is further supported by its capacity to reduce energy intake and enhance energy expenditure, contributing to its potential weight loss effects [28]. This functionality is the basis for developing FGF21-based therapeutics and dual agonists, such as those combining FGF21 with glucagon-like peptide-1 (GLP-1), which are being explored for their efficacy in treating obesity and related metabolic disorders [28].

Obesity is recognized as a complex metabolic disease that can predispose individuals to additional health issues, including type 2 diabetes mellitus (T2DM) and cardiovascular diseases (CVD). Our study’s positive correlation between FGF21 levels and BMI reinforces the concept that elevated FGF21 may indicate increased metabolic stress associated with higher body fat.

In a study conducted on monozygotic twins, it has been estimated that 40% of the variability in serum FGF21 levels can be attributed to genetic factors, suggesting a significant contribution of genetic inheritance to this characteristic [30]. However, this finding also implies a more powerful influence of environmental factors in explaining the observed differences in FGF21 concentrations among individuals, highlighting the substantial impact of external factors on this biological marker [30]. Serum FGF21 levels are recognized for exhibiting considerable variability even within the healthy population [31]. As demonstrated in this study, factors influencing FGF21 levels may include unknown variables, such as serum chloride level, diastolic blood pressure value, and well-known factors, such as BMI.

Given these insights into FGF21 variability and recognizing its correlation with BMI, we aimed to investigate how the four distinct BMI groups differ regarding echocardiographic parameters. Our findings reveal significant differences in the dimensions of left ventricular wall thickness and vena cava inferior (VCI) across the four BMI groups. Specifically, we observed a notable difference in left ventricular (LV) wall thickness between patients with BMI < 25 kg/m^2^ and those with BMI > 35 kg/m^2^. This suggests that as BMI increases, there is a tendency toward greater wall thickness in the LV, which aligns with the literature [32,33]. Similarly, our study found significant differences in VCI dimensions between patients in the BMI range of 25–30 kg/m^2^ and those in the BMI range of 30–35 kg/m^2^. This variation might reflect changes in hemodynamic parameters and venous return associated with increasing BMI, contributing to different echocardiographic patterns [34].

Obesity is known to induce chronic low-grade inflammation [35]. This inflammatory environment can affect the liver and contribute to liver enzyme alterations, including elevated AST. A higher BMI is associated with an increased likelihood of hepatic steatosis [36]. As the liver changes in response to excess fat accumulation, it may lead to inflammation and cellular stress, influencing AST levels. AST is also a marker of myocyte necrosis [37]. In the first group, there are 12 patients with myocardial infarction; in the second, 8; in the third, 15; and in the last 10; thus, the average value of AST is higher in the third group. Because acute coronary syndromes are characterized by a degree of inflammation, in the same way, the distribution of mean leukocytes can be explained with higher values in the third group. For the erythrocyte sedimentation rate, a higher value is observed in the last group, with a statistical difference between obesity groups three and four.

Furthermore, our results indicate a linear and significant increase in triglyceride levels with higher BMI. Potential mechanisms include insulin resistance—particularly abdominal obesity is associated with insulin resistance, which in turn increases hepatic triglyceride synthesis; increased dietary intake—diets high in carbohydrates and fats can contribute to elevated triglycerides in obese individuals; and inflammation—obesity is associated with chronic low-grade inflammation, which can affect lipid metabolism [38].

Our study, which included patients with acute coronary syndromes, revealed that serum FGF21 levels were elevated compared to those reported in the literature for healthy subjects. This finding aligns with a recent meta-analysis involving 29,156 individuals from 30 studies, which demonstrated that serum FGF21 concentrations were significantly higher in patients with CVD (*p* < 0.001), particularly in those with CAD and hypertension (*p* < 0.001). The meta-analysis highlighted that elevated serum FGF21 levels are strongly associated with an increased risk of CVDs, potentially independent of vascular parameters [39]. However, our study presents a divergence from the meta-analysis findings concerning hypertension. While the meta-analysis did not observe any significant association between FGF21 and systolic blood pressure (summary r = 0.13) or diastolic blood pressure, we found a correlation between FGF21 and diastolic blood pressure in our cohort. This divergence suggests that the interaction between FGF21 and blood pressure might be more complex than previously understood and could differ among patient populations. Compared to normal subjects, the elevation of FGF21 levels in our study’s CVD patients supports the notion that FGF21 could be a valuable biomarker for assessing CVD risk.

In our study, we provided evidence that the circulating level of FGF21 was higher in patients with unstable angina, similar to the findings of Cheng J and colleagues [40]. However, the FGF21 level was lower than in those with myocardial infarction. Additionally, consistent with Cheng J’s study [40], there was a correlation between the FGF21 level and creatine kinase, but no correlation with troponin T. FGF21 levels are elevated in patients with myocardial infarction compared with healthy individuals, as demonstrated by several studies [41,42]. Furthermore, recent research indicates a strong association between the no-reflow (NR) phenomenon, a key indicator of poor prognosis in acute STEMI patients, and elevated FGF21 levels [43].

The most reliable correlations between FGF21 levels and clinical parameters are consistently linked to lipid profile markers (triglycerides, HDL, and LDL), liver-related indicators (liver fat content, γ-GT, AST), and factors associated with insulin sensitivity/resistance (HOMA-IR, fasting insulin) [27]. Additionally, associations with adiponectin, high blood pressure, BMI, age, and kidney function further highlight the complex interplay between FGF21 and diverse physiological factors under metabolic strain [27]. Our findings revealed significant positive associations between FGF21 levels, body mass index, diastolic blood pressure, and chloride. These results underscore the intricate regulatory mechanisms governing FGF21 dynamics, challenging conventional associations and necessitating a nuanced interpretation of its role in diverse pathophysiological contexts.

Moreover, our regression analyses identified robust associations between FGF21 and uric acid, chloride, and creatine kinase MB, shedding light on specific pathophysiological factors influencing FGF21 levels in our cohort. These associations may indicate potential connections between purine metabolism, salt balance, myocardial injury, and serum FGF21 levels within the studied population. The r^2^ values in the regression analyses are relatively low, indicating that the selected variables explain a small proportion of the variance in FGF21 levels. This indeed suggests that the predictive value of these variables is limited.

From a biological perspective, uric acid (UA) can exhibit pro-oxidative and anti-oxidative properties [44,45]. Under physiological conditions, UA has antioxidant effects primarily as an oxygen radical scavenger, neutralizing superoxide anions, hydroxyl groups, singlet oxygen, and other reactive substances in vivo [46]. This serves to protect the cardiovascular system from oxidative stress damage. However, UA acts as a pro-oxidant in conditions with high UA levels or low levels of other antioxidants [46]. Its oxidative effects involve mediating the immune response after cell injury [47], enhancing pro-inflammatory immune activation [48], and promoting low-density lipoprotein oxidation [49], smooth muscle cell proliferation, platelet activation, and adhesion [50]. Consequently, UA exerts oxidative and antioxidant effects at different concentrations [46]. In cardiovascular disease, UA is considered a ‘double-edged sword’ with beneficial and detrimental effects on cardiovascular health [51,52]. In the context of our study, the correlation between FGF21 and uric acid may be explained by the reciprocal influence of these two factors in metabolic response and oxidative stress. FGF21, known for its role in metabolic regulation and protection against oxidative stress, could interact with uric acid levels depending on the study subjects’ specific physiological or pathological state.

## 5. Conclusions

The objective of this study was to investigate the relationship between FGF21 levels and anthropometric, clinical, biochemical, and echocardiographic parameters in patients diagnosed with acute coronary syndromes, with a specific focus on the correlation between FGF21 levels and the degree of obesity. Our findings highlight the extensive variability in circulating FGF21 levels, ranging from below the detection limit to significantly elevated concentrations, which align with the existing literature.

Our study observed a well-established correlation between FGF21 levels and BMI. This correlation is consistent with the existing literature and aligns with expected findings. Additionally, we identified new correlations between FGF21 levels and serum chloride and diastolic blood pressure, which have not been previously emphasized. Regression analyses identified robust associations between FGF21 and uric acid, chloride, and creatine kinase MB, suggesting specific pathophysiological factors influencing FGF21 levels in our cohort. Importantly, our analysis showed no statistically significant differences in FGF21 levels between the four BMI groups or within diagnostic categories.

Given the study’s limitations, including a relatively low number of enrolled patients, a heterogeneous patient population, and FGF21 levels measurable in only 55 patients, the statistical analyses should be interpreted cautiously. These limitations reduce the generalizability and robustness of the conclusions drawn. The study provides only a snapshot of FGF21 levels at a single time point, and longitudinal observations are necessary to fully understand the biomarker’s behavior and its implications over time.

## Figures and Tables

**Table 1:** Clinical and laboratory data from our study with 80 patients were classified into four groups based on their BMI: Group 1 (18.5–24.9 kg/m^2^), Group 2 (25–29.9 kg/m^2^), Group 3 (30–34.9 kg/m^2)^, and Group 4 (>35 kg/m^2^). Each group’s mean and standard deviation for various parameters is provided. Statistical analysis using one-way ANOVA was conducted to compare the means across the four groups. The parameters are divided into five groups. The first group provides information on gender, age, degree of hypertension, smoking status, family history of cardiovascular disease, previous cardiovascular treatments, and diagnosis at admission. The second group includes height, weight, waist circumference, and BMI. The third group contains clinical parameters such as systolic blood pressure, diastolic blood pressure, heart rate, and oxygen saturation. The fourth group includes echocardiographic measurements, and the last includes laboratory measurements. The *p*-values from the ANOVA tests indicate the statistical significance of differences among the groups for each parameter.

**Parameter**	**Group 1** BMI 18.5–24.9 kg/m^2^	**Group 2** BMI 25–29.9 kg/m^2^	**Group 3** BMI 30–34.9 kg/m^2^	**Group 4** BMI >35 kg/m^2^	
**Number of Patients**	20	20	20	20	
**Sex (M/F)**	13/7	12/8	16/4	7/13	
**Age (years, mean ± SD)**	60.45 ± 8.84	60.50 ± 8.60	61.20 ± 8.06	58.05 ± 7.77	
**Hypertension (HTN)**					
No	4	3	3	3	
- HTN 1	2	1	1	0	
- HTN 2	6	3	8	3	
- HTN 3	8	13	8	14	
**Tobacco Use**					
No	11	12	5	12	
Yes	7	7	9	5	
Ex-smoker	2	1	6	3	
**Family History of CV** **Any CV conditions**					
No	16	10	8	7	
Yes	4	10	12	13	
**Previous CV Treatment**					
No	7	4	6	4	
Yes	13	16	14	16	
**Admission Diagnosis**					
STEMI	10	7	10	6	
NSTEMI	2	1	5	4	
Unstable Angina	8	12	5	10	
**Anthropometric Measurements**					
**Parameter**	**Group 1**	**Group 2**	**Group 3**	**Group 4**	***p* Value**
**Height (m)**	1.68 ± 0.06	1.68 ± 0.08	1.69 ± 0.10	1.63 ± 0.08	0.10
**Weight (kg)**	66.85 ± 6.70	81.15 ± 9.46	91.70 ± 11.50	105.45 ± 14.07	<0.001
**Waist Circumference (cm)**	85.90 ± 13.89	98.85 ± 6.22	110.40 ± 15.83	117.35 ± 11.10	<0.001
**BMI (kg/m^2^)**	23.44 ± 1.64	28.51 ± 0.94	31.75 ± 1.02	39.51 ± 4.66	<0.001
**Clinical Measurements**					
**Parameter**	**Group 1**	**Group 2**	**Group 3**	**Group 4**	***p* Value**
**Systolic Blood Pressure (mmHg)**	126.75 ± 21.96	135.55 ± 28.39	130.25 ± 19.89	136.25 ± 23.61	0.53
**Diastolic Blood Pressure (mmHg)**	77.25 ± 11.05	79.70 ± 14.94	75.25 ± 10.19	80.75 ± 14.35	0.52
**Heart Rate (bpm)**	77.50 ± 9.93	72.10 ± 11.61	76.60 ± 12.53	80.25 ± 13.42	0.19
**Oxygen Saturation (%)**	97.09 ± 0.80	96.83 ± 2.13	97.47 ± 1.25	97.31 ± 0.95	0.49
**Echocardiographic Parameters**					
**Parameter**	**Group 1**	**Group 2**	**Group 3**	**Group 4**	***p* Value**
**Ascending Aorta (mm)**	34.28 ± 2.25	34.53 ± 4.47	34.11 ± 3.03	34.11 ± 3.04	0.97
**Interventricular Septum (mm)**	11.29 ± 1.27	12.72 ± 1.34	12.24 ± 1.57	13.26 ± 2.03	0.002
**Posterior Wall of the Left Ventricle (mm)**	10.59 ± 1.36	12.21 ± 1.32	11.63 ± 1.23	12.25 ± 1.63	<0.001
**Left Ventricle in Systole (mm)**	35.89 ± 6.74	36.25 ± 6.28	35.24 ± 5.50	40.45 ± 9.74	0.10
**Left Ventricle in Diastole (mm)**	46.22 ± 7.71	48.31 ± 5.82	48.17 ± 4.68	49.71 ± 7.34	0.41
**Left Atrium (mm)**	36.26 ± 3.26	38.68 ± 5.59	36.18 ± 3.45	39.01 ± 5.74	0.10
**Right Ventricle (mm)**	33.16 ± 2.88	32.52 ± 5.33	32.36 ± 3.06	32.92 ± 6.83	0.95
**Right Atrium (mm)**	35.33 ± 2.84	36.99 ± 6.03	35.23 ± 4.08	35.99 ± 4.97	0.61
**Inferior Vena Cava (mm)**	15.47 ± 2.73	13.91 ± 2.04	16.03 ± 2.69	15.73 ± 2.57	0.04
**Ejection Fraction (%)**	46.45 ± 9.98	49.50 ± 8.41	48.00 ± 5.71	47.00 ± 8.49	0.66
**Laboratory Measurements**					
**Parameter**	**Group 1**	**Group 2**	**Group 3**	**Group 4**	***p* Value**
**Uric Acid (mg/dL)**	7.66 ± 10.98	5.75 ± 2.02	5.52 ± 1.25	6.22 ± 1.85	0.63
**Alanine Aminotransferase (U/L)**	28.75 ± 21.28	26.55 ± 21.67	35.45 ± 17.28	25.05 ± 9.90	0.28
**Aspartate Aminotransferase (U/L)**	93.55 ± 101.96	63.05 ± 104.94	130.80 ± 141.68	41.75 ± 40.86	0.04
**Cholesterol (mg/dL)**	173.75 ± 49.50	186.95 ± 45.33	183.45 ± 46.22	197.24 ± 69.85	0.58
**Creatinine (mg/dL)**	0.90 ± 0.21	0.96 ± 0.37	0.91 ± 0.25	0.99 ± 0.35	0.78
**Glucose (mg/dL)**	138.95 ± 75.68	109.50 ± 33.64	126.65 ± 45.50	148.40 ± 59.87	0.41
**HbA1C (%)**	6.54 ± 1.42 (n = 17)	6.25 ± 0.97 (n = 14)	7.03 ± 1.09 (n = 14)	7.26 ± 1.58 (n = 16)	0.15
**HDL Cholesterol (mg/dL)**	48.87 ± 13.88	43.65 ± 10.29	41.91 ± 14.94	42.96 ± 10.14	0.30
**Chloride (mmol/L)**	103.79 ± 4.49 (n = 14)	103.36 ± 4.30 (n = 14)	102.69 ± 4.02 (n = 13)	103.71 ± 3.60 (n = 14)	0.89
**Potassium (mmol/L)**	4.40 ± 0.42	4.32 ± 0.56	4.39 ± 0.55	4.59 ± 0.618	0.42
**Sodium (mmol/L)**	137.50 ± 3.72	138.25 ± 2.55	136.40 ± 4.51	138.00 ± 3.62	0.39
**LDL Cholesterol (mg/dL)**	114.10 ± 39.53	117.99 ± 43.79	112.35 ± 45.42	131.25 ± 49.25	0.53
**C-Reactive Protein (mg/L)**	14.44 ± 16.24 (n = 9)	12.12 ± 10.07 (n = 4)	25.90 ± 28.81 (n = 11)	20.18 ± 18.54 (n = 9)	0.59
**Triglycerides (mg/dL)**	101.85 ± 47.14	123.75 ± 74.32	140.80 ± 87.09	183.55 ± 100.86	0.01
**Urea (mg/dL)**	36.35 ± 13.62	45.05 ± 15.74	41.00 ± 12.45	42.20 ± 21.61	0.40
**Fibrinogen (mg/dL)**	362.63 ± 103.17 (n = 8)	329.50 ± 153.44 (n = 6)	391.25 ± 124.69 (n = 8)	390.60 ± 173.88 (n = 5)	0.83
**Hemoglobin (g/dl)**	14.08 ± 1.33	13.48 ± 1.49	14.23 ± 2.33	12.99 ± 1.56	0.09
**Leukocytes (×10^3^/µL)**	10.24 ± 3.18	9.04 ± 2.31	11.65 ± 3.10	9.69 ± 2.44	0.03
**Platelets (×10^3^/µL)**	251.00 ± 59.21	252.05 ± 58.91	262.00 ± 48.35	252.30 ± 56.37	0.91
**ESR (mm/h)**	18.55 ± 12.06	18.80 ± 14.94	16.30 ± 15.03	32.75 ± 25.66	0.01
**PRO-BNP (pg/mL)**	3517.60 ± 6816.05 (n = 10)	977.64 ± 1396.77 (n = 11)	1864.42 ± 2399.19 (n = 12)	2888.00 ± 4205.95 (n = 5)	0.53
**High-Sensitivity Troponin T (pg/mL)**	2959.22 ± 2066.45 (n = 9)	1938.37 ± 3179.78 (n = 11)	2263.18 ± 2399.91 (n = 12)	681.60 ± 653.86 (n = 5)	0.42
**Creatine Kinase (U/L)**	169.50 ± 105.75 (n = 6)	274.20 ± 172.30 (n = 5)	285.00 ± 342.67 (n = 7)	62.33 ± 52.00 (n = 3)	0.48
**FGF 21 (pg/mL)**	238.98 ± 303.66 (n = 14)	296.78 ± 394.29 (n = 13)	373.77 ± 386.72 (n = 12)	449.94 ± 430.17 (n = 16)	0.47

**Table 2:** Comparison of FGF21 levels in the 55 samples, categorized by diagnosis.

SCA	Case (n, %)	FGF21 (pg/mL) Means	*p*
STEMI	21 (38.18%)	245.72	0.1
NSTEMI	9 (16.36%)	257.89	
Unstable Angina	25 (45.45%)	456.28	
Total	55 (100%)	342.42	

**Table 3:** Correlations of FGF 21 levels. Kendall’s tau-b coefficient. *p* < 0.05. (*) indicates statistically significant values at the *p* < 0.05 level.

Parameter	τ	*p*
BMI	0.193 *	0.038
Diastolic Blood Pressure	0.214 *	0.034
Chloride	0.269 *	0.025

**Table 4:** Significant simple linear regression between FGF21 and various parameters.

Parameter	Mean	Std Deviation	F	*p*	R^2^
Uric Acid	6.56	6.71	5.208	0.027	0.089
Chloride	103.33	4.42	5.048	0.031	0.129
Creatine Kinase	239.00	258.22	7.857	0.019	0.440

## Data Availability

The datasets generated during and/or analyzed during the current study are available from the corresponding author upon reasonable request.
